# “*When I Breastfeed, It Feels as if my Soul Leaves the Body*”: Maternal Capabilities for Healthy Child Growth in Rural Southeastern Tanzania

**DOI:** 10.3390/ijerph17176215

**Published:** 2020-08-27

**Authors:** Zaina Mchome, Sepideh Yousefzadeh, Ajay Bailey, Hinke Haisma

**Affiliations:** 1 Population Research Centre, Faculty of Spatial Sciences, University of Groningen, P.O. Box 800, Landleven 1, 9747 AD Groningen, The Netherlands; h.h.haisma@rug.nl; 2 National Institute for Medical Research, Mwanza Centre, P.O. Box 1462, 33104 Mwanza, Tanzania; 3 Campus Friesland, University of Groningen, P.O. Box 3, Wirdumerdijk 34, 8911 CE Leeuwarden, The Netherlands; s.yousefzadeh@rug.nl; 4 Department of Human Geography and Spatial Planning, International Development Studies, Utrecht University, P.O. Box 80.115, Princetonlaan 8a 3584 CB Utrecht, The Netherlands; a.bailey@uu.nl; 5 Transdisciplinary Center for Qualitative Methods, Manipal Academy of Higher Education, Manipal 576104, India; 6 International Union for Nutrition Sciences Task Force Toward Multi-dimensional Indicators of Child Growth and Development, 10 Cambridge Court, 210 Shepherds Bush Road, London W6 7NJ, UK

**Keywords:** capability approach, capability framework for child growth, maternal capabilities, child growth, breastfeeding, growth monitoring, Tanzania, ethnography

## Abstract

The burden of childhood stunting in Tanzania is persistently high, even in high food-producing regions. This calls for a paradigm shift in Child Growth Monitoring (CGM) to a multi-dimensional approach that also includes the contextual information of an individual child and her/his caregivers. To contribute to the further development of CGM to reflect local contexts, we engaged the Capability Framework for Child Growth (CFCG) to identify maternal capabilities for ensuring healthy child growth. Ethnographic fieldwork was conducted in Southeastern Tanzania using in-depth interviews, key informant interviews, participant observation, and focus group discussions with caregivers for under-fives. Three maternal capabilities for healthy child growth emerged: (1) being able to feed children, (2) being able to control and make decisions on farm products and income, and (3) being able to ensure access to medical care. Mothers’ capability to feed children was challenged by being overburdened by farm and domestic work, and gendered patterns in childcare. Patriarchal cultural norms restricted women’s control of farm products and decision-making on household purchases. The CFCG could give direction to the paradigm shift needed for child growth monitoring, as it goes beyond biometric measures, and considers mothers’ real opportunities for achieving healthy child growth.

## 1. Introduction

The problem of poor growth among children under five in different regions in Africa is becoming increasingly acute [1,2]. The current evidence indicates that in African countries, 58.8 million (33%) under-five children are stunted and about 14 million are wasted, and 9.5 million children were overweight in 2018, up from 6.6 million in 2000 [3]. In the Sub-Saharan region, 33% of under-five children are stunted, 2.3% are wasted, and 3.5% are overweight [1]. In Eastern Africa, 35.2% of under-five children are stunted, 1.6% are wasted, and 4.7% are overweight (Ibid). Tanzania is no exception: it is among the 10 countries with the highest burden of childhood stunting (i.e., 34%). Tanzania also has the double burden of malnutrition, with 28% of women and 4% of under-five children suffering from overweight and obesity [4].

To prevent children from becoming malnourished, a number of initiatives have been implemented at both global and local levels. Child growth monitoring (CGM) is one of the initiatives designed to enable timely identification of (physical) growth faltering and appropriate intervention. CGM has been incorporated into the health policies of most countries, including Tanzania, and is reflected in child care practices [5]. For decades, the discourse on child growth has been dominated by the biomedical paradigm, which constructs CGM as “the process of following the growth rate of a child in comparison to a standard by periodic anthropometric measures” [1]. In this discourse, a mono-dimensional approach that focuses only on anthropometric indicators has been used not only to identify at-risk children, but also to develop interventions to promote healthy growth. It has become the norm for health workers and public health programmers to evaluate the growth of under-five children and to intervene based on information about changes in weight and height [5,6,7,8,9].

Child growth indicators have been used to monitor progress in meeting the Millennium Development Goals, particularly MDG 4—reduction in under-five mortality rate—and MDG1—poverty reduction [10]. However, CGM has been criticized for not consistently improving the health and nutrition of under-five children [8,11]. While efforts were made in Tanzania to reach the MDG targets, the rates of childhood malnutrition, and particularly of stunting, declined only slightly during the MDG era, [12]—i.e., from 48% in 1999 to 43% in 2010. The current trend also shows that Tanzania is still far from achieving the Sustainable Development Goals (SDGs)—specifically goal 2 of “zero hunger” that aims to end all forms of malnutrition by 2030—as it still has unacceptably high (33%) levels of childhood undernutrition [4]. Surprisingly, the burden of stunting in Tanzania is also persistently high (above 40%) even in high food-producing regions [4]. Relying on anthropometric indicators to understand child growth provides only a partial answer to both “who” and “why” questions [7]. While they are important tools for identifying who is exhibiting healthy growth and who is not, anthropometric indicators provide neither further information about the socioeconomic or cultural characteristics of the children, their caregivers, or their communities nor a deeper understanding of why some children are malnourished (ibid). The paradoxical question of why the prevalence of childhood stunting is high in a context of plenty calls for an approach to assess child growth that captures not only anthropometric outcomes, but also contextual information on individual children and their caregivers. This information is important, as children (and their caregivers) lead their lives under a variety of conditions [13] that shape their growth differently.

In Tanzania, several scientists and scholars have contributed to our understanding of contextual determinants of poor growth, which include child malnutrition at the household and societal level [14,15,16,17,18]. However, as most of these studies use biomedical–anthropometric-indicators, they hardly capture the complexity of the growth of specific children [7]. In other words, they do not examine the contextual factors that lead to diversities among children and caregivers in terms of what they can or cannot do to achieve healthy growth. Information on the local realities that contribute to healthy child growth is crucial, as it would help health workers provide appropriate advice to caregivers during CGM, and it could be used in policy-making processes aimed at allocating resources more equitably and reducing inequalities (ibid).

Assessing well-being by considering what individuals can or cannot do with their resources is emphasized in Amartya Sen’s capabilities approach from the field of welfare economics [19]. In this paper, we use concepts from Sen’s capability approach to shed light on maternal capabilities that contribute to healthy child growth in Morogoro region. We discuss how each of the capabilities is influenced by individual and contextual factors, and how they, in turn, shape healthy child growth. Our study is one of the few empirical studies [20] that applies the capabilities approach to understanding healthy child growth.

## 2. Capabilities Approach: A Theoretical Framework

First developed in the work of Amartya Sen [21], the capabilities approach was used to explain why people starve when there is plenty of food in their locality. The key thesis of the capabilities approach is that when assessing an individual’s well-being, the primary focus should be on what people are effectively able to do and be, i.e., their capabilities. Capabilities represent the “real opportunities” of an individual to do or achieve something [22]. Drawing conclusions about well-being based on people’s capabilities is crucial, as individuals differ in their abilities to transform the available resources into achieving what “they value” [19], such as “healthy child growth” [6,7]). The diversity of people’s capabilities is shaped by the independent or interactive interplay of conversion factors that enable or disable the conversion of resources into functionings [22]. Conversion factors are categorized into three groups: personal, social, and environmental [22]. *Personal conversion factors* are internal to the person (e.g., physical condition, age, sex, and reading skills). *Social conversion factors* stem from the wider society where a person lives (e.g., public policies, social norms, power relations related to class, gender). *Environmental conversion factors* represent physical characteristics surrounding the individual (e.g., climate, housing conditions, and road conditions). According to the capabilities approach, in the process of achieving what they value, people exercise agency, which is the freedom and the ability to choose from the available options to pursue one’s goals [19]. From the capabilities approach perspective, an individual’s values and choices are shaped by the specific socio-cultural context in which s/he lives [22]. Accordingly, people make choices that are embedded in their context-specific values (ibid).

**Figure 1:**
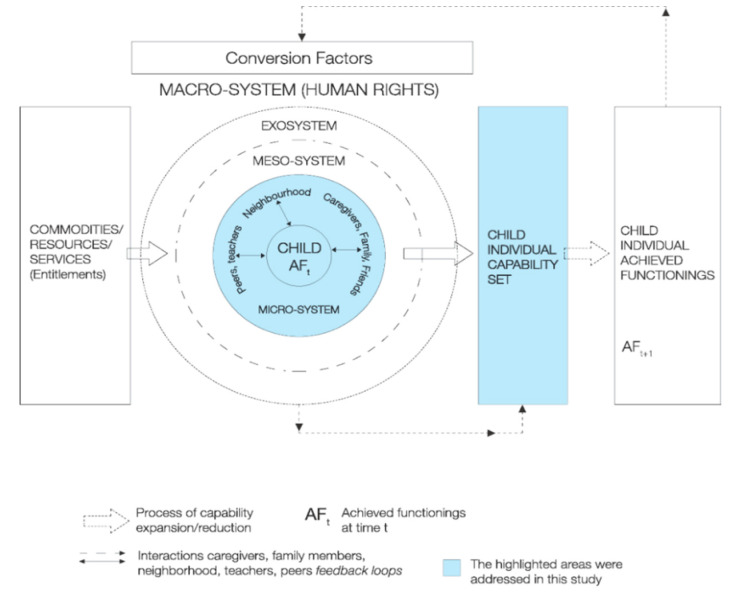
Constitutive elements of capabilities framework for child growth (Yousefzadeh et al., [7]). Reprinted by permission from Springer Nature Customer Service Centre GmbH: Springer Nature, Child Indicators Research, (A Capability Approach to Child Growth, Yousefzadeh, S.; Biggeri, M.; Arciprete, C.; Haisma, H.), Copyright (2018).

In the context of nutrition and child growth, the capabilities approach suggests the need for a multi-dimensional approach to assessing child growth that goes beyond applying anthropometric measures to evaluate what individual caregivers are effectively able to do and be, and their choices for achieving healthy child growth. As Haisma et al. [6] clearly articulated, child growth is complex and multi-dimensional, and efforts to evaluate it should reflect that complexity, rather than being narrowly focused. Recently, a Capability Framework for Child Growth has been developed that reflects this multi-dimensional perspective on child growth [7]. This study applies the CFCG (for more details, see Figure 1) to identify capabilities for achieving healthy child growth.

## 3. Methods

### 3.1. Study Design

This paper draws on data gathered during three months of ethnographic fieldwork conducted in Malangali, a rural village in Kilosa District, Southeastern Tanzania. The principal investigator, accompanied by a research assistant, both women with a background in medical sociology, lived in the village for three months (in two separate periods) to conduct the fieldwork. From July to September 2015, the researchers conducted a household census, collected anthropometric measurements of under-five children, and conducted 19 focus group discussions. From August to September 2016, we conducted 30 in-depth interviews and five key informant interviews. Participant observations were made throughout the fieldwork period. The findings in this paper are mainly drawn from data collected through participant observations, focus group discussions, and in-depth interviews with biological mothers and fathers of under-five children, irrespective of their nutritional status, and key informant interviews with community health workers (CHWs) and traditional birth attendants (TBAs). As defined by the World Health Organization, the TBAs are persons who assists the mothers during childbirth, and have initially acquired their skills by delivering babies themselves or through apprenticeship to other traditional birth attendants [23].

### 3.2. Study Setting

The fieldwork setting of this research has been described elsewhere [24], but, in short, Morogoro region is considered one of Tanzania’s “food baskets”—i.e., high food-producing regions—but has a relatively high prevalence of stunting (33%) among under-five children [4]. The rural location of Kilosa District, which has a high prevalence of infant malnutrition and anemia in a context of plenty, make it an appealing choice for examining the capabilities that contribute to healthy child growth. The major income-generating activity of residents in this district is small-scale farming. Others engage in petty trade, formal/skilled employment, and self-employment through different forms of unskilled manual labor. While most of the agricultural activities we observed were conducted by men and women alike with a shared aim of feeding the family, harvesting and storing the harvests were depicted as male responsibilities. Threshing maize (*kupukuchua mahindi*), taking care of children, and doing household chores were characterized as female tasks.

### 3.3. Recruitment of Participants

Recruitment of participants for key informant interviews: Key informants included community health workers (CHWs) and Traditional Birth Attendants (TBAs). Both CHWs and TBAs are important local health experts, consulted by parents about health and growth issues of their child. In the study village—just like in other villages in Tanzania—there are two CHWs; they are involved in routine child growth monitoring services in the village, and are linked to a dispensary. TBAs are consulted by mothers in relation to maternal issues. The two CHWs were recruited through the village chairman’s support. The CHWs were then requested to link the researcher with TBAs, the second group of key informants in this research.

Recruitment of participants for in-depth interviews and focus group discussions: The participants in in-depth interviews and focus group discussions were recruited through the help of CHWs and local leaders, and through the researcher’s social networks. Given their role in providing growth monitoring services to under-five children in the community, and thus having good knowledge of parents with under-five children, CHWs facilitated the identification of some mothers and fathers of under-five children for IDIs from the wider community. Some of the participants for IDIs were identified by the researcher from the wider community through individual social networks developed while living in the field, while others were identified from the clinics’ attendees at the child growth monitoring clinic during participant observation. In this case, during her visits to the growth monitoring clinics, the researcher—in consultation with the CHW—explained the purpose of her presence at the clinic to mothers, and identified mothers for in-depth interviews after their children’s growth was assessed. Furthermore, the participants in the focus group were purposively recruited from the community with the help of the local leaders and other relevant gatekeepers. The researcher requested the local leaders to mobilize a number of potential individuals for recruitment for participation in the focus group discussions. As the principal researcher was living in the community, she visited the individuals in their settings, asking them about their children, and used screening questions to determine the individual’s eligibility for participation. The screening ensured that each participant had a biological child under five years old and is a permanent resident of the village. To avoid recruiting people of the same social network, different entry points were used. After it was established that the individuals met the study’s eligibility criteria, the person was given detailed information about the study, and was asked whether she/he was willing to take part. For individuals who expressed interest and willingness to participate, appointments for discussion were made based on their availability. During data collection, there reached a time when further probing in the interviews and FGDs did not reveal new codes. At this stage, data saturation was reached and we decided to stop recruitment.

### 3.4. Data Collection

We conducted in-depth interviews to gain insight into caregivers’ personal views and experiences of the capabilities and contexts underlying the growth of their individual children. The focus Group Discussions aimed to capture general opinions on parents’ capabilities contributing to healthy growth in their under-five children. For interviews and focus-group discussions, topic guides with open-ended questions and probes that covered various topics, including perceptions of child growth, contextual factors underlying child growth, child feeding practices, and experiences with growth monitoring services, were developed using the CFCG and piloted. The issues related to parental capabilities, conversion factors, and agency relevant to healthy child growth were captured using questions on the parents’ daily responsibilities in the family, perceived qualities of a good mother/father in relation to her/his child, mothers’ and fathers’ roles in promoting healthy growth, what parents can do to make their children grow well, what parents think they would need to make their children grow well, the environments that contribute to parents’ ability and/or inability to provide good care for their children, and stories about moments when they wanted to take care of their children but could not. All focus group discussions and in-depth interviews were conducted in Swahili, audio-recorded using a digital recorder, and later transcribed *verbatim*. Most interviews were arranged in the participants’ homes at times convenient to them. The focus group discussions were conducted in different venues, including school classrooms (after school hours), and the principal researcher’s and participants’ home compounds.

In order to gain insights into the socio-cultural and economic contexts in which parents/caregivers and their children live, the principal researcher set up a household and lived in the study community for the entire period of the fieldwork. While in the field, she took part in the village life and participated in daily activities. Passive participant observation was conducted during day-to-day activities such as visiting and chatting with neighbours at their homes, attending village meetings, churches and burial ceremonies. More active participant observation involved cooking with women, participating in the intimate household level child care activities such as feeding and carrying the baby around, accompanying mothers to the child growth monitoring clinic, to the market for groceries, joining women to fetching water from the boreholes, accompanying them to pick greens from gardens near their house and to the farms at a few hours walking distance. Walking around between the houses/neighbourhoods allowed the researcher to observe the daily preparation of food, timing of feeding, whether people preferred or avoided certain types of food, eating arrangements, and groups of men and women busy in farms, et cetera. The researcher’s house location, in particular, placed her in a setting where she could easily meet and observe villagers—particularly early in the morning, and in the evening—as they passed to and from their farms. Furthermore, living and working in the field while pregnant and later together with her newborn, narrowed the distance between the researcher and the women who participated in the research, something that motivated them to freely share their stories. Additionally, her stay in the field and the fact that most of the in-depth interviews were conducted in participants’ homes enabled her to witness the poor condition that some of children and their parents were living in and thus contributed to her understanding of the construction of healthy child growth in this context. Throughout the fieldwork, the researcher wrote fieldnotes on day-to-day encounters, and kept a reflective diary.

### 3.5. Data Analysis

Data analysis started in the field, as the researchers documented the themes that emerged from day-to-day encounters. This enabled researchers to go deeper into the subsequent focus group discussions/interviews. As Strauss and Corbin [25] emphasize, in ethnography, data collection and analysis are not distinct phases, as an initial analysis of the information gathered in the early phases of fieldwork shapes the future course of the work in a reiterative process. To preserve linguistic authenticity, all transcripts were analyzed in Swahili. The analysis took place at two levels. In the first circle of coding, the inductive and deductive codes were developed. First, a series of inductive codes was developed based on the principles of Grounded Theory [25], whereby theoretical insights emerge from the data rather than being pre-specified. Inductive codes were determined through a close reading and re-reading of transcripts. Second, the deductive coding was performed based on theoretical components of the capability approach that informed the data collection topic guides and maternal capabilities could be identified. The inductive and deductive codes were discussed and refined by the researcher and the fourth author. In the next circle of coding, the codes were categorized into groups of themes—as described in Hennink, Hutter, and Bailey [26]—which reflected maternal capabilities for healthy child growth. All transcripts were imported to and coded using NVivo 11 software (QSR International Pty Ltd., Doncaster, Australia). Additional data from field notes were used to clarify and expand the perspectives of the caregivers in relation to maternal capabilities for healthy child growth.

### 3.6. Positionality

The researcher was a mother and was pregnant at the time of the first round of fieldwork. During the second round, she had two children, and the newborn was with her in the field. The researcher’s role as a mother encouraged the women to trust her and to share their stories about how they navigate the challenges they face in ensuring the healthy growth of their children. Although the researcher and the research assistant were younger than some of the participants, the principal investigator being a mother masked the disadvantage of appearing young to some of the participants, as it brought them into the peer group of adults who were able to empathize with the caregivers’ stories.

### 3.7. Ethical Issues

The study was approved by the Research Ethics Committee at the Faculty of Spatial Sciences of the University of Groningen in Groningen, the Netherlands and the Tanzanian Ministry of Health, Community Development, Gender, Elderly and Children through the Medical Research Coordinating Committee (MRCC) in Dar es Salaam, Tanzania (NIMR/HQ/R.8a/Vol.IX/1974). Additionally, permission was granted by the regional, district, and village leaderships prior to the commencement of the research activities. Full information was provided to participants verbally and as a written copy in Swahili, and written/thumbprint consent was obtained. Participants’ confidentiality and anonymity were ensured by conducting the discussions/interviews in private locations, and removing all identifiers from the interview transcripts prior to data analysis. In the quotations in this paper, pseudonyms are used to protect the participants’ identities.

## 4. Results

Three maternal capabilities emerged from the analysis of the caregivers’ narratives on healthy child growth: (1) being able to feed their children, (2) being able to control and make decisions on farm products and income, and (3) being able to ensure access to medical care. We also discuss each of these capabilities by showing how they are influenced by contextual and/or individual factors and caregivers’ agency, and how they shape the promotion of healthy child growth. Despite the presence of different types of participants in our sample, our analysis did not find any significant differences among them in perspectives regarding maternal capabilities for healthy child growth.

## 5. The Capability to Feed

In the study setting, the mother is depicted as the main caretaker (*mlezi*) of under-five children, and is thus considered responsible for the health and growth of her children. When describing the qualities of a good mother that helps a child grow well, participants mentioned “providing good care to children.” From the participants’ perspective, “good child care” mainly entailed breastfeeding a child, and cooking and feeding a child nutritious food at the right times. A number of conversion factors were noted that could enable or disable the mothers to achieve the ability to feed their children. We grouped these factors into two categories: (i) personal conversion factors and (ii) social conversion factors.

### 5.1. Personal Conversion Factors and the Mother’s Capability to Feed

Paternal capabilities and behaviors: The analysis shows that a mother’s capability to feed a child in utero and after birth interacts with those of the father, i.e., her partner. For instance, fathers “being able to earn,” and “being able to ensure that mothers are healthy and nourished during pregnancy and lactation period” became endowments for mothers to provide care, thus promoting healthy child growth. The following quote illustrates how a father’s capability to ensure good nutrition to a mother during pregnancy results in a “good”-sized newborn child.

*When a mother is pregnant there are particular nutritious foods that she is supposed to use [eat] so that she promotes the health of the baby in her womb. You see, when a mother gives birth, you will find that the baby is in good health. When the baby is born in good health you will hear people say, ‘Someone has given birth to a big baby, the baby is bigger than her mother.’ The baby is big to the extent that when s/he is taken to be weighed you find that it has three or four kilos, you see! That means that a father took a good care of his wife when pregnant. (FGD-#03-Father)*

Additionally, the partner’s behavior could compromise a woman’s resources or constrain her ability to feed her children. For example, engaging in alcoholism, polygamy, extra-marital sexual relationships, or pregnancy denial emerged as factors that could compromise a father in his role as parent and partner, and leave the mother with extra productive and reproductive responsibilities to compensate for his unavailability as a husband and caregiver. This, in turn, affected the mother’s functioning, including her ability to adequately feed her children. Mothers reported that some men were misusing meager family resources, neglecting their children, and spending most of their time away from home. They added that if the father was neglecting his children or refusing to provide for the family, the mother could exercise agency by seeking support from local leaders, who often intervened and made the fathers provide economic support for his children’s daily needs, particularly food and medical care. A number of mothers said they had reported their partner to local leaders to make them provide for the family.

*I told him (partner) that the child has been found with UTI and malaria, and that the cost is 3000 shillings. He said, ‘I do not have that 3000 shillings.’ I then decided to report him to the sub-village leader. The leader helped me to make him provide that money. I then paid the lady (nurse). (IDI-#05-Mother)*

Our observations and interviews indicate that women were overburdened by agricultural and domestic work, particularly during the rainy season. Mothers (primary caregivers) reported spending most of their day performing farm work—even during pregnancy—and then having to do domestic chores, including cooking for the family, when they returned home. Mothers’ struggles to perform multiple roles left them exhausted, and affected their capability to provide good care, particularly adequate and timely feeding. Participants often ascribed poor child feeding to mothers having to occupy multiple roles.

*When returning from the farm work, you are tired, but the household chores await you (majukumu yanakusubiri). You are supposed to fetch water, cook, feed children, wash children, wash clothes, and clean the house. Ideally, a child is supposed to eat three times a day, in the morning, in the afternoon, and in the evening. That is the mother’s good care. But now, I return from farm work at 3 pm, it takes a lot of time to prepare mboga (leafy greens) and cook ugali. Eventually, my children eat late. In most cases, they end up eating twice a day. (IDI-#01-Mother)*

*At times, I overstay there [in the field] and return home late. I start cooking, but find that by the time I finish cooking, it is already late evening. They (children) end up eating just a single meal per day, as you cannot return early from the farm. (IDI-#02-Mother)*

Moreover, spending long hours doing farm work was reported to contribute to early weaning among mothers, as in addition to making it hard for mothers to find time to breastfeed, it often caused mothers to skip lunch, which could, in turn, cause them to have insufficient breast milk or energy to breastfeed.

*We usually go to farms early in the morning and return home at 2.00 p.m. At times, we even lack a chance to eat lunch. You only come to get ugali (stiff porridge) in the evening, around 4:00 p.m. That is when you return from the farm, you start cooking, and you finish around 6:00 p.m. That is the only meal you have for the day. While working in the farm, you are starving, but the baby breastfeeds on you the whole day. Thus, both you and the baby starve. When I breastfeed, it feels as if my soul leaves the body. So, you say, aah, it is better to stop breastfeeding her/him so that we can both eat ugali. Or you say, it is better to introduce food to him/her early so that s/he does not heavily breastfeed on you. (KII-#03-TBA)*

To be able to feed their children while doing farm work, mothers often went to the fields with their babies, and took porridge and snacks with them. Although this improved mothers’ capability to (breast) feed, it negatively compromised children’s rest and good sleep, as, in most cases, the babies slept on the ground. The babies were also exposed to cold weather and rain during the wet season, and sun and dust during the dry season. Mothers often carried their babies on their backs when doing farm work, which was tiring for both the mother and the child.

*Working with the babies in the farm is not pleasant at all. The farm environment is for work, not for resting. [...]. You find that a child cries so much, s/he becomes exhausted as most of the time s/he is tied on her/his mother’s back. S/he must be tired. A child needs to relax, play a little bit, and sleep. But in the farms, s/he lacks that. (IDI-#03-Father)*

Mothers pointed to the partner’s support as one of the important contributors to their ability to feed their children while they were doing farm work. While a few mothers reported receiving support from their partner in carrying the baby so that they could cook for the family after returning from work, only one man admitted to helping his wife with domestic chores, including cooking and feeding under-five children. In the study community, it was noted that a father could help his wife by hiring a housemaid to do household chores and to take care of under-five children while she was doing farm work. However, the majority of the men lacked the financial means to hire help. The failure of many fathers to provide sufficient support to the mother in doing domestic work and providing direct care to under-five children was ascribed to patriarchy, which framed domestic works and taking care of children as “feminine responsibilities.”

*Most of men in our village are patriarchal (wana mfumo dume). They leave the burden [of child care] to mothers. You find that a mother has a lot of activities to do, and is still supposed to look after children. [...] With that situation, instead of preparing food that has nutrients proper for healthy growth of her child, a mother decides to feed her child kiporo (dinner left-overs) so that s/he does not bother her with his/her hunger. That’s why you find that a child becomes malnourished as s/he is not being provided with food suitable for her/his age. (IDI-#07-Father)*

Additionally, maternal social networks were noted to be important conversion factors that promoted a mother’s capability to provide good care, including feeding her children while away doing farm work. Mothers who were living in close proximity to important others like grandmothers and those who had good relationships with their neighbors, explained that they could leave their children at their grandmother’s or their neighbor’s while they were doing farm work. Some reported leaving their weaned babies at home under their older siblings’ care, particularly during the weekends. Clarifying how having good relationships with neighbors and having friends around enhances mothers’ ability to provide good care, including feeding their children, one participant during interview said:

*I have best friends (maswahiba) with whom my child is comfortable. They like to stay with my child. One of the neighbors, who is with my son as we speak… I used to leave my child at her place, go to the farm and return late in the evening, say at 3 pm. I even do not leave behind any food for him (child), because what they will eat, my child also eats. She (friend) is so helpful. (IDI-#03-Mother)*

### 5.2. Socio-Cultural Conversion Factors and Mother’s Capability to Feed

A mother’s ability to (breast)feed her infant was also shaped by socio-cultural conversion factors, including (i) the cultural schema that a new pregnancy during lactation period spoils a mother’s breast milk [27]; (ii) the cultural schema that an infant’s persistent crying at night indicates that the child is “born with hunger” (*mtoto amezaliwa na njaa*), and therefore cannot be satisfied by the mother’s breast milk; (iii) the belief that a child’s reduced interest in breastfeeding is caused by threats from *mdudu* (unseen evil spirit) residing in the mother’s body. These beliefs were not held by the mothers alone, but were widespread in the community, including among the TBAs and CHWs who supported the mothers in the feeding process.

*When a woman becomes pregnant prematurely, she can’t continue to breastfeed while knowing that she has a baby in her womb. She has to stop breastfeeding, short of that, the baby will contract excessive diarrhea, which will weaken her and make her growth falter. […] It is important to immediately stop breastfeeding completely when you notice that you are pregnant, even if the baby is three or five months old. (KII-#02-TBA)*

*People in our community have come to know over the years that when an infant cries much, it means that s/he is born with hunger. Thus, when a baby reaches three weeks, one month or two months, you find that s/he [caregiver] starts feeding her/him some ‘uji’ (porridge). (KII-#01-CHW)*

*Other children are denied breastfeeding by the evil spirits. When a mother has an evil spirit in her body, the child is being threatened by that evil spirit that you have. So, when a child is scared s/he avoids coming closer to you. […] when that happens, a mother decides to give the baby porridge or cow’s milk. (FGD-#03-Mother)*

### 5.3. Capability to Control and make Decisions on Farm Produce and Income

In the study setting, farming was a joint responsibility of men and women. However, many mothers reported that, because they lacked the capability to control and make decisions about the farm products and the earnings from selling those products—which was culturally prescribed as a male responsibility—their children’s capabilities to be adequately fed and to get timely and proper treatment when they fell ill were limited. The analysis showed that women tended to be more concerned with keeping agricultural products for family needs, while men were market-oriented, and prioritized selling the harvests to get money to finance other forms of consumption, including buying assets and alcohol and having extra-marital relations. The findings revealed that men were even more likely to sideline women from managing farm products if women did not participate in farm work. It was reported that, in such cases, men habitually sold the farm crops without their partner’s knowledge. In many households, men’s tendencies to sell the family food reserves and to squander the income obtained led to food insecurity, financial constraints, and conflict between couples.

To enhance their capability to make decisions about farm income in order to better care for their children, mothers developed different coping strategies. Many decided to rent land from the village farm or from a private farm, and to cultivate this separate farm rather than the family farm, while others used their extra time to work for wages at other people’s farms to satisfy their nutritional needs, as well as those of their children.

*In our context, men control farm income. Thus, a woman decides to cultivate her own farm, and her man cultivates his own farm. When you harvest, you have your own income, and the man will have his own income (IDI-#04-Mother).*

Mothers explained that to stop their husband from misappropriating the crops and sidelining them from controlling the agricultural produce, which was particularly likely to occur when they did not participate in farm work, they worked while pregnant almost up to their due date, and resumed working at least 40–60 days after birth.

*You work in the farm from the first month of pregnancy till when you are almost due. In the ninth month if you feel that you are so tired, you may rest for one month, and then give birth. When you give birth, you continue working. […] If you refuse to go to the farm, when he harvests, he will not involve you with the products. He could even decide to sell all the crops in the farm. You will just hear that your partner has sold the crops and has married another wife. Women are now conscious! She goes to work in the farm even when she has a little infant. This way, men hardly find a good reason to misappropriate the crops. (KII-#03-Traditional birth attendant)*

Mothers reported that doing farm work even when pregnant, working on their own farm, and engaging in wage labor enhanced their capability to control farm products and earnings. This enabled them to make decisions about daily household purchases, and to prioritize appropriate care for the optimal growth of their children. However, their engagement with extra farm work added to their heavy workload, which affected their capability to rest well, and further reduced their capability to provide good care to their children. Similarly, in some cases, mothers reported delaying seeking health care for their sick children because they were busy with extra farm work, which jeopardized the children’s capability to receive timely medical care. Women who had complications during their pregnancy or childbirth faced more inequalities due to the loss of work and income.

### 5.4. Capability to Assure Access to and Utilization of Medical Care

In the study setting, mothers were depicted as the first people in the family to recognize that a child was sick or had special needs, and they were expected to ensure that their children got good and timely medical care. In most cases, when a mother realized that a child was ill, she immediately told her husband, not only because it was the norm to do so, but because she was dependent on her husband’s economic support in seeking medical care for her ill child.

*Usually, when a child is sick, a mother is the first person who will notice. She will then inform you (a father) that this little one is sick. Even if a child cannot speak, the mother has the capacity to recognize an ill condition in her child. By being close to a child, she can just tell if something is wrong in his/her (baby’s) health” (FGD-FOU-#03)*

Ensuring a child’s access to and utilization of medical care emerged as an important capability for mothers related to promoting healthy child growth. The analysis indicated that a mother’s capability to ensure that her children got proper medical care when ill was affected by the interactive interplay of multiple conversion factors, notably having a low income coupled with a lack of economic support from her partner when their children had health emergencies. Although many mothers confirmed that they had the capability to make decisions about care-seeking themselves, the process of executing their decisions was hindered by their lack of money to cover the costs related to medical care. A considerable number of mothers lamented that their partner tended to take their children’s illnesses lightly, and provided little or delayed support when asked to pay for their children’s medical care. The situation was worse for mothers who were in polygynous unions, as their husband was often not at home, or neglected their children when they were ill and needed treatment.

In their narratives about their children’s access to medical care, mothers clarified that their partner rarely helped them take the children to routine child care clinics, as such roles were culturally perceived as feminine. In this context, if a father took his child for CGM, he would be ridiculed by community members, particularly fellow men, and perceived as being under his wife’s control or charmed (*ametawaliwa na mke wake/amelishwa limbwata*).

*Men in our context do not take children to the clinic. They feel shy (laughs). He will be laughed at by his colleagues. They will ask him, ‘Why do you take the child to the clinic? Are you controlled by your wife? (umetawaliwa na mkeo?).’ That is what most of them (men) fear. (IDI-#01-Mother)*

*Many men lack confidence. People talk about being charmed by a wife. That’s why one may ask himself, ‘Should I take the child to the (CGM) clinic? No way, this is my wife’s role.’ So, he leaves it up to his wife. (IDI-#05-father)*

Other conversion factors that challenged the mother’s capability to ensure access to medical care included (1) the lack of a health facility in the village; (2) distance to the health facility; (3) impassable roads, particularly during the wet season; (4) the lack of public transport facilities; (5) shortages of drugs in most public health facilities. The study village, like most areas in Kilosa District, has a flat topography and is surrounded by rivers and farms. Thus, during heavy rains, a large part of the village floods. The poor road conditions in the wet season, coupled with the poor transport system, limited caregivers’ ability to travel to nearby health facilities (approximately 3–5 km away), particularly when they had maternal and child-health-related emergencies. Additionally, despite the policy requirement in Tanzania that the under-five children receive free medical services from the government facilities, caregivers had to pay out-of-pocket to cover diagnostic visits, medicine, and hospital stays. The interplay of those conversion factors was reported to result in delays in seeking health care for children, self-medication of children’s illnesses, particularly through the use of drugs leftover from previous treatments, and reliance on drugs from small shops in the village and traditional remedies. Confirming the mothers’ concerns about the lack of health facilities, geographical factors, and poor roads, among other conversion factors that contributed to their inability to ensure access to medical care, which in turn affected child growth, one of the fathers during FGDs stated:

*The lack of a dispensary (health facility) in this village is a big challenge towards our efforts to ensure that our children get better health care. We are greatly affected. Geographically, our area is troublesome during rainy season. We also lack good roads which could enable someone to rush someone to the nearby hospital in times of health emergencies. If we could have a dispensary in our village, it would be much better. […] So, if a child falls sick, and if you are not careful, you may be surprised that the child dies at home. (IDI-#04-Father).*

Although mothers’ ability to ensure children’s access to medical care seemed to be challenged by myriad conversion factors, including their dependency on the father as the head of the household, our analysis showed that within their narrow space of agency, mothers found ways to navigate medical care options for their ill children. Many mothers said they relied on community health workers (CHWs) when their children were ill and they lacked the means to pay for treatment. Services provided by the community health workers included, but were not limited to, counseling on child growth and health issues, referrals to health facilities, and, in some cases, prescribing drugs and medical treatments to both caregivers and their under-five children. Others said they consulted traditional birth attendants (for delivery and child health issues) and traditional healers and remedies (for the treatment of children’s illnesses). Mothers were also entitled to receive loans to pay for medications for their children from health workers in nearby health facilities and the small privately owned drug shops (*maduka ya dawa*) in the community. Beyond the formal and informal health service providers, neighbors, relatives, and local leaders played vital roles as direct and indirect advisors to mothers. Friends and neighbors also assisted by lending money or organizing transportation. Additionally, if a mother was being neglected by her partner, she was entitled to receive support from her parents and in-laws, or to have the local leaders’ support in making the partner cover the child’s medical costs.

*You find that the child is ill and needs immediate treatment, but the father is not around or he tells you that he does not have money. What will you do? You must use your brain. If you have a friend, you go and ask her for a help or a loan so as you take your child to the hospital, with the agreement of refunding her later. (IDI-#04-Mother)*

*One day my son was feverish, I could not sleep. I was alone as his father does not sleep here. When the morning came, I informed his father about the child’s condition. He told me to go to the drug shop for medication. I decided to take the child to the hospital for diagnosis. I only had 1000 shillings, while the diagnosis costs 3000 shillings. So I asked the nurse to help me (give medication on loan), she trusted me and told me to bring the remaining 2000 shillings later. When I returned home, I told him (partner) that the child has been diagnosed with UTI and malaria, and that the cost is 3000 shillings. He said, ‘I do not have that 3000 shillings’. I then decided to report him to the sub-village leader. The leader helped me to make him provide that money. I then paid the lady (nurse). (IDI-#05-Mother)*

## 6. Discussion

In this capability analysis of our ethnography on child growth, we found that healthy growth among under-five children was shaped by three specific maternal capabilities: 1. being able to feed their children; 2. being able to control and make decisions on farm produce and income; 3. being able to ensure access to medical care. We also show how these capabilities and conversion factors are interlinked.

### 6.1. Being Able to Feed

Mothers—the primary caregivers—were overburdened by farm and domestic work, which limited their capability to (breast) feed their children adequately. It is known that when mothers are overburdened by domestic and farm work, their children are more likely to be undernourished [16,18,28,29]. Overwork among mothers has been linked to inadequate breastfeeding and complementary feeding elsewhere as well, including in Bangladesh [30], Rwanda [31], Viet Nam [32], and Tanzania [15,16,18,33]. However, to our knowledge, mothers lacking the strength to breastfeed (indicating their exhaustion) and the time to eat as a result of farming activities have not been mentioned before. This was exemplified by the quote, “it feels as if my soul leaves the body” which illustrates the severity of the burden of breastfeeding while managing farming responsibilities.

Based on the aforementioned findings, we argue that the prominent role of agriculture in rural Tanzania and women’s engagement in agricultural activities partly explain: (1) why the rural poor in Tanzania have higher levels of stunting than urban residents; (2) why stunting occurs even in areas with relatively high food availability, including in the Morogoro region, which was the study setting. Our study findings call for interventions to address the multiple pressures on women to help them overcome the work and time demands that greatly reduce their capability to feed their children. Ahishikiye et al. [31] suggested reducing women’s domestic and agricultural burdens, and encouraged them to engage more in home-based income-generating activities so they can allocate more time to childcare. This suggestion does not acknowledge the problem of men not sharing productive and reproductive responsibilities. We would caution that interventions to address women’s challenges related to farm work need further thinking to ensure that they do not hamper women’s agency. We believe that since women have traditionally been engaged in the farming business for decades, detaching them from agricultural production may cause them to lose their agency, as they lack their own income. We argue along the same lines as Hodgson [34] that initiatives to address issues related to women’s engagement with farm work should strive to achieve gender complementarity. In this context, parents should be encouraged to complement each other in fulfilling their roles and responsibilities in ways that promote the healthy growth of their children. Thus, fathers should become more involved in domestic affairs in order to complement mothers’ efforts to provide care to the children. However, more operational research is needed to determine how fathers could be successfully engaged in domestic affairs, including providing direct care to under-five children. Additionally, our findings point to the need for nutritionists to promote and advocate exclusive breastfeeding while taking into account that mothers’ capability to breastfeed is related to (a) mothers’ productive roles and (b) fathers’ productive and reproductive roles.

In addition to women’s heavy workload, the early introduction of complementary feeding was shown to be influenced by cultural schemas, especially those framing sexual activity and the view of a new pregnancy during lactation period as harmful to the growth of the baby. Similar schemas have also been reported by previous ethnographic studies conducted in Tanzania [27,35,36]. Our findings point to the need for open discussions between health workers and mothers about cultural issues related to breastfeeding, and for health workers to be more respectful of cultural models. Both pre-service and in-service training for health service providers—including TBAs and CHWs—on cultural competence [37,38] could help bridge the gap between biomedical and local explanations of breastfeeding, and thus improve parent–health worker communication [39]. As suggested by Mchome et al. [27], health workers could also focus on addressing the existing misconceptions around breastfeeding, such as those related to sexuality and a new pregnancy during the lactation period in their health promotion activities.

Mothers navigated the different social support systems—social networks—that were available to them to provide what they found to be better care. Mothers were assisted in the ability to feed, for example, by grandmothers, older children, and neighbors in looking after and feeding their under-five children when they were away doing farm work. The role of social support in enhancing a mother’s capability to feed her children has also reported in other studies conducted elsewhere [31,40,41,42,43]. While the importance of social networks in enhancing a mother’s capability to feed her children while away from home is crucial, it must be emphasized that the extended social support cannot replace the father’s role in promoting healthy child growth. Additionally, the practice of leaving weaned children in the care of an older sibling, as observed in this study, is worth noting, as it may limit the caregiver’s attention to younger children, and expose them to poor care [16,18,33,44,45].

### 6.2. Being Able to Control and Make Decisions on Farm Produce and Income

Another factor that mediated mothers’ capabilities related to child growth was the household power structure within which child care occurred. The patriarchal society and its associated cultural norms restricted women’s control of farm produce and decision-making about household purchases. Mothers reported that their ability to feed and ensure access to medical care for their children interacted with the fathers’ ability to provide for the family as the head of the family and the breadwinner. As a result, the mothers could not buy appropriate food for their children or take their children to hospital without their husband’s financial support—which was noted to be scarce due to men’s behavior, including alcoholism, polygamy, and extra-marital sexual relationships. The relationship between women’s access to and control over financial assets and growth outcomes for their children is also reported in studies elsewhere [15,46,47,48]. As Kariuki et al. [49] have observed, when women have more control over the family’s financial resources, a larger proportion of the income is allocated to children’s basic needs. The findings in this study point to the need to improve women’s earnings and economic justice at the household level. However, we caution that changing patriarchal norms is not easy, and has taken several decades in Western societies. Thus, in this context, rather than economically empowering women only—which has shown to increase tensions among parents, and even to lead to different forms of violence against women [50]—we suggest that both men and women in the study setting should be economically empowered. Through this strategy, both men and women will be able to have control over their income, thereby strengthening each other’s ability to achieve healthy child growth. Since our study findings confirm that parents’ ability to ensure healthy child growth interact and are interdependent, the economic empowerment strategy suggested in this paper should have a gender training component in order to raise fathers’ awareness that caring for children and providing for the family are shared responsibilities. Fathers should also be motivated to cease harmful behaviors that affect their wives and children.

### 6.3. Being Able to Ensure Access to Medical Care

Mothers mentioned poor paternal support in providing direct care to children, including taking children to routine CGM clinics, as it was constructed as a feminine role. Although we understand the importance of promoting the engagement of men in child care, including feeding, as recommended by [31,51,52], we argue that the interventions that promote men’s engagement in maternal and child health care in areas similar to the study setting may not succeed unless they address cultural norms that deter men from providing direct care to under-five children, including attending child care clinics, and focusing on inequalities that are intertwined with gender relations.

Additionally, neighbors, drug store attendants, community health workers, and professional health workers played vital roles in facilitating mothers’ ability to ensure access to and utilization of medical care for their children. The caregivers’ reported reliance on small drug shops and CHWs for accessing medical care for themselves and their children is alarming, as (i) the CHWs are not professional health care providers, and (ii) the drug shops in the community are operated by unqualified attendants who offer a variety of medications, mostly without prescription [24]. The practice of relying on these workers may fuel self-medication practices, which have many adverse effects [53]. To avoid harm that may result from the misuse of the CHWs’ power, we recommend routine monitoring of the operation of CHWs, and occasional refresher trainings for CHWs that remind them of the need to adhere to their specific roles and their ethical obligations. The general community should also be informed of the roles of CHWs, including that they are not professional health care providers.

## 7. Conclusions

Through the use of CFCG framework, the findings show that healthy growth—a functioning at an early age—is a result of the interactive interplay of maternal capabilities, among others. The lived experiences of the mothers in the study area indicated that child growth outcomes are plural, and go beyond the indicators at the child level to include factors external to the child, such as the parents’ (mothers’) capabilities. Our findings are useful in rethinking child growth monitoring, as has also been suggested by [5]. Based on our study findings, we advocate for a CGM paradigm shift to ensure the healthy growth and development of under-five children. Specifically, we recommend that the practice of growth monitoring engages a multidimensional approach that goes beyond the determinants level and biometric measures, and considers the caregivers’ ability to ensure healthy child growth. We would argue, in line with Chakraborty [20], that instead of defining and assessing child growth as an anthropometric outcome only, efforts should be made to understand child growth and to intervene in childhood malnutrition while taking into account the parents’ capabilities. Moreover, if these capabilities are declining, the focus should be on promoting the caregivers’ awareness of what they value (i.e., their capabilities). For example, in the study village, the mothers’ awareness of their ability to feed their children, to control their family’s resources and to make decisions about their family’s income, and to ensure access to medical care was bounded by unequal gender roles, their financial constraints and dependency on their husband, their agency, and gendered norms and structures. From the findings, addressing culturally bound practices that affect child health are key to healthy child growth. Efforts should focus on addressing the unequal division of labor by educating the community that domestic tasks are the responsibility of both men and women. Similarly, efforts are needed to reduce women’s burdens in agricultural production, and to improve their access to resources and decision-making about family income. Based on the study’s findings, we recommend policies that protect and advance women’s rights by providing them with more opportunities so that they can choose what they value most—in this case, providing good care to their children.
